# Comparison of productivity and quality of bacterial nanocellulose synthesized using culture media based on seven sugars from biomass

**DOI:** 10.1111/1751-7915.13401

**Published:** 2019-03-25

**Authors:** Genqiang Chen, Guochao Wu, Lin Chen, Wei Wang, Feng F. Hong, Leif J. Jönsson

**Affiliations:** ^1^ College of Chemistry, Chemical Engineering and Biotechnology Donghua University Shanghai 201620 China; ^2^ Department of Chemistry Umeå University SE‐901 87 Umeå Sweden

## Abstract

*Komagataeibacter xylinus* ATCC 23770 was statically cultivated in eight culture media based on different carbon sources, viz. seven biomass‐derived sugars and one sugar mixture. The productivity and quality of the bacterial nanocellulose (BNC) produced in the different media were compared. Highest volumetric productivity, yield on consumed sugar, viscometric degree of polymerization (DP_v_, 4350–4400) and thermal stability were achieved using media based on glucose or maltose. Growth in media based on xylose, mannose or galactose resulted in lower volumetric productivity and DP_v_, but in larger fibril diameter and higher crystallinity (76–78%). Growth in medium based on a synthetic sugar mixture resembling the composition of a lignocellulosic hydrolysate promoted BNC productivity and yield, but decreased fibril diameter, DP_v_, crystallinity and thermal stability. This work shows that volumetric productivity, yield and properties of BNC are highly affected by the carbon source, and indicates how industrially relevant sugar mixtures would affect these characteristics.

## Introduction

Bacterial nanocellulose (BNC), also known as microbial cellulose or bacterial cellulose, is a high value‐added biomaterial synthesized mainly by acetic acid bacteria. It possesses unique structure and properties, such as nanofibrillar structure, high degree of polymerization and high mechanical strength, which endow BNC with great potential in the areas of textile manufacturing, fibre‐based paper and packaging products, food industry, biomedical materials, and advanced functional bionanocomposites (Gama *et al*., 2012; Lin *et al*., 2013; Lee *et al*., 2014; Mohite and Patil, 2014). However, many potential applications for BNC are restricted by its relatively high price. The high price is in part due to the high cost of the culture medium including the carbon source.

Previous investigations have addressed production of BNC from agro‐industrial by‐products and cellulosic residues such as konjac glucomannan (Hong and Qiu, 2008), wheat straw (Hong *et al*., 2011; Chen *et al*., 2013), waste fibre sludge (Cavka *et al*., 2013; Chen *et al*., 2017), spruce wood residues (Guo *et al*., 2016), waste cotton textile (Hong *et al*., 2012; Guo *et al*., 2013) and sweet sorghum bagasse (Chen *et al*., 2018a,2018b). Media based on different feedstocks contain many different sugars. The exact effects of these different sugars on the production and quality of BNC are not well understood, and need to be further investigated. It is important to identify low‐value carbon sources that are converted with high productivity to BNC of high quality.

There have been some previous efforts aiming at investigating BNC productivity using media based on different sugars (Keshk and Sameshima, 2005; Dahman, *et al*., 2010; Mikkelsen *et al*., 2009). However, these studies were not comprehensive, and the quality of the BNC produced was not thoroughly investigated. Only four biomass‐derived sugars were studied, and very limited quality characterization was performed. For instance, there was no characterization of the degree of polymerization (DP). Quality characterization would include features such as DP, crystallinity, fibril diameter and thermal stability. Therefore, it is important to perform a comprehensive investigation of the effects of media based on different sugars on the productivity and quality of BNC using the same bacterial strain and the same cultivation conditions.

In this study, media based on eight different carbon sources were investigated. These carbon sources included seven common sugars derived from plant biomass, and one sugar mixture designed to resemble the composition of a lignocellulosic hydrolysate. The sugars included glucose, xylose, mannose, arabinose and galactose, all of which are common monosaccharide sugars derived from lignocellulosic biomass. The sugar mixture designed to resemble a lignocellulosic hydrolysate included these five monosaccharides. Sucrose, which is a common disaccharide obtained from sugar cane and sugar beet, was also included. The eighth carbon source was maltose, which is a common disaccharide obtained from starch. BNC quantity and quality as well as pH and residual sugar concentrations were monitored. The analyses covered surface morphology, fibril diameter, fourier‐transform infrared (FTIR) spectroscopy, DP, crystallinity and thermal stability.

## Results and discussion

### Comparison of BNC production from eight carbon sources

**Figure 1:**
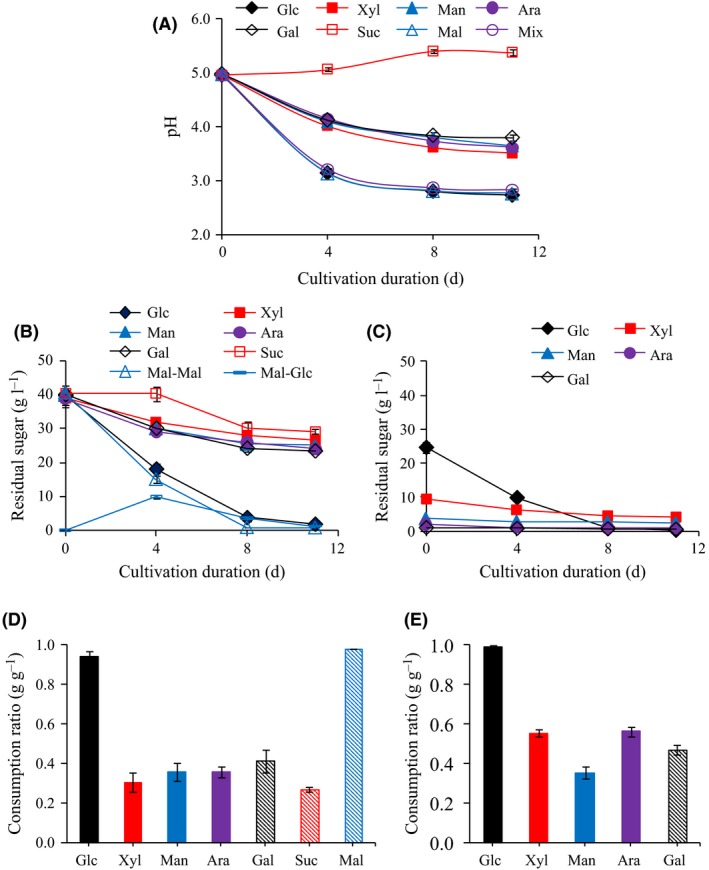
Time‐course of pH (A), sugar concentrations (B and C) and sugar consumption ratios (D and E) for cultures with eight different carbon sources. C and E show residual sugar concentrations and sugar consumption ratios in medium with a sugar mixture. Maltose would be partially hydrolysed to glucose, and therefore, the concentrations of maltose and glucose in the media are shown as Mal‐Mal and Mal‐Glc (B).

**Figure 2:**
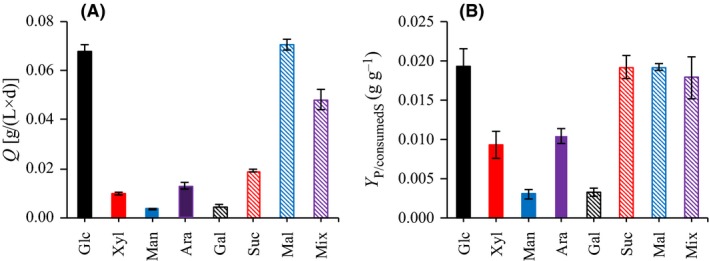
Volumetric bacterial nanocellulose (BNC) productivity (A) and yield of BNC on consumed sugar (B) from cultures with eight carbon sources. ‘P’ and ‘S’ refer to product and sugar respectively.

The bacterial strain studied was *Komagataeibacter xylinus* ATCC 23770. Static cultivations were carried out for 14 days. In the first series of experiments, the effects of the eight carbon sources on pH evolution (Fig. 1), sugar consumption (Fig. 1), and BNC productivity and yield (Fig. 2) were compared.

In media based on glucose, maltose and the sugar mixture, the pH decreased quickly to around 3 in 4 days, and then, in the next 7 days, it decreased slowly (Fig. 1A). In media based on xylose, mannose, arabinose or galactose, the decrease in pH was smoother (Fig. 1A). Among the seven media based on single sugars, glucose and maltose were consumed most rapidly and were exhausted after 11 days (Fig. 1B and D). The presence of glucose in the medium with maltose as carbon source was due to the hydrolysis of maltose (Fig. 1B). The other sugars were consumed much slower than glucose and maltose, and the consumption ratios were less than half of those of glucose and maltose (Fig. 1B and D). For sucrose, there was even a 4‐day lag phase (Fig. 1B). Thus, the two disaccharides in the study, sucrose and maltose, were metabolized with very different rate. In the medium with the sugar mixture (Fig. 1C and E), the consumption ratios of almost all the sugars were higher than in the media with single sugars, except for mannose, which exhibited similar consumption ratios (Fig. 1D and E). The consumption ratios of xylose and arabinose were enhanced by more than 0.20 g g^−1^, while the consumption ratios of glucose and galactose were enhanced by 0.05–0.06 g g^−1^. An evaluation using t‐test showed that these enhancements were statistically significant (*P* < 0.05).

The volumetric BNC productivity and the BNC yield on consumed sugars are shown in Fig. 2. As seen in Fig. 2A, the volumetric BNC productivity for media with glucose and maltose [around 0.07 g/(L×d)] was significantly higher than for the other media (*P* < 0.05). Cultures with mixed sugar medium exhibited the third highest volumetric BNC productivity (Fig. 2A). The volumetric BNC productivity of the mixed sugar cultures [0.048 g/(L×d)] was 1.1 times higher than the sum of the productivities of single sugar media with 24 g l^−1^ glucose, 9 g l^−1^ xylose, 4 g l^−1^ mannose, 2 g l^−1^ arabinose and 1 g l^−1^ galactose [theoretically 0.044 g/(L×d)] (significant difference with *P* < 0.05). The theoretical value, 0.044 g/(L×d), was calculated based on the assumption that the contribution to the productivity of each of the single sugars, i.e. glucose, xylose, mannose, arabinose and galactose, was ideally linear to that of the initial concentration of the sugar. Thus, the volumetric BNC productivities of cultures with single sugars at a concentration of 40 g l^−1^ (Fig. 2A) were used for the calculation of the theoretical value. The enhanced BNC productivity of the mixed sugar cultures compared to single sugar cultures should be related to the accelerated sugar consumption observed for the mixed sugar cultures (Fig. 1E). The volumetric BNC productivity of sucrose‐based cultures was much lower than that of maltose‐based cultures (significant difference with *P* < 0.05). This was expected considering the obvious difference in assimilation between sucrose and maltose (Fig. 1B).

The BNC yield on consumed sugar for cultures with sucrose medium was similar to those of cultures with glucose and maltose media (around 0.019 g g^−1^) (Fig. 2B), although the BNC productivity for cultures with sucrose medium was far lower than for cultures with glucose and maltose media (Fig. 2A). Furthermore, the BNC yield on consumed sugar for cultures with the sugar mixture was almost as high as the corresponding values for cultures with glucose and maltose media. The BNC yield on consumed sugar for cultures with the sugar mixture (0.018 g g^−1^) was 1.2 times higher (significant difference with *P* < 0.05) than the sum of the theoretical yields for cultures with media based on 24 g l^−1^ glucose, 9 g l^−1^ xylose, 4 g l^−1^ mannose, 2 g l^−1^ arabinose and 1 g l^−1^ galactose (which was 0.015 g g^−1^, calculated as indicated above). For cultures based on xylose or arabinose, the BNC yields on consumed sugar were lower than for cultures based on glucose, maltose, sucrose, or the sugar mixture, and for cultures based on mannose or galactose they were even lower (significant differences with *P* < 0.01) (Fig. 2B).

The BNC yields on consumed sugar are comparable to those found in other studies. Kurosumi *et al*. (2009) studied BNC production by *K. xylinus* NBRC 13693 in synthetic media based on glucose, fructose and sucrose. For removal of microbial contaminants, pellicles were washed successively with water, 2% (w/v) NaOH, 2% (v/v) acetic acid and water. Depending on the type of sugar, the BNC yield on consumed sugar was in the range 0.003–0.021 g g^−1^ (Kurosumi *et al*., 2009). In the study of Singhsa *et al*. (2018), BNC was produced from the *K. xylinus* strains TISTR 086, TISTR 428, TISTR 975, and TISTR 1011, and with media based on glucose, fructose, lactose, maltitol, sucralose or xylitol. The crude BNC was washed with 2% (w/v) NaOH at 80°C for 1 h and then with deionized water. The BNC yield on consumed sugar was in the range 0.002–0.031 g g^−1^ depending on the bacterial strain and the carbon source (Singhsa *et al*., 2018).

The enhanced BNC productivity and yield on consumed sugar observed for cultures with the sugar mixture agree with the previous study of Dahman *et al*. (2010). They studied the effect of using a sugar mixture (based on the sugar composition of hydrolysates of wheat straw, corn fibre and distillers grain) for BNC production and showed enhanced BNC yield on consumed sugar compared to that of single sugar cultures (Dahman *et al*., 2010). Differences between the two studies include that the current study is the first to show increased sugar consumption ratios for cultures based on individual sugars in the sugar mixture and that Dahman *et al*. (2010) used shaking cultivation rather than static cultivation. Moreover, in contrast to previous work in the field (Dahman *et al*., 2010), the current study covers the effects of individual sugars and a sugar mixture on the properties of the BNC.

### Morphology of BNC

The macroscopical appearance of the BNC from cultures based on the eight carbon sources was similar, as gelatinous pellicles were formed on the surface of the culture medium. This observation agrees with previous reports (Watanabe *et al*., 1998; Bi *et al*., 2014).

**Figure 3:**
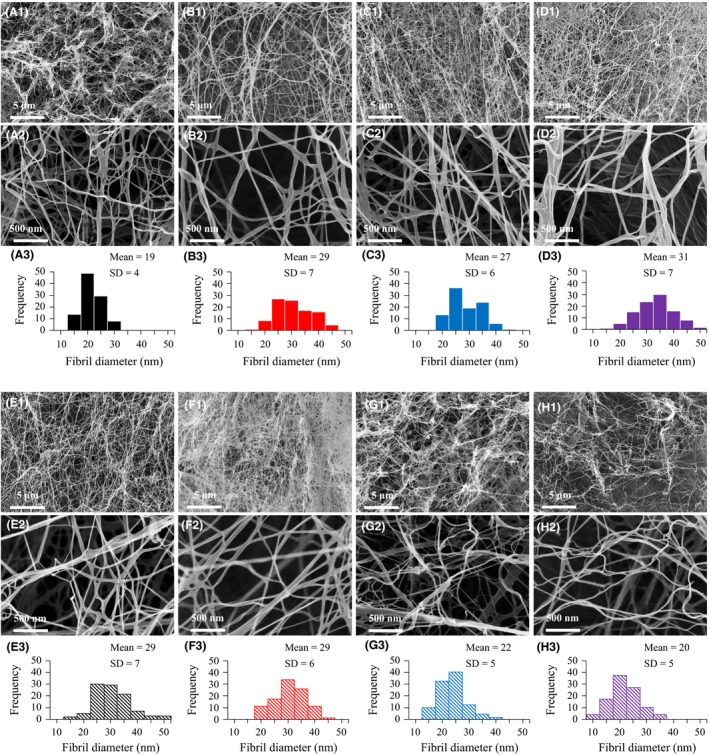
Scanning electron microscopy micrographs and fibril diameter of bacterial nanocellulose (BNC) produced from eight carbon sources. The eight carbon sources for the BNC were glucose (A1–A3), xylose (B1–B3), mannose (C1–C3), arabinose (D1–D3), galactose (E1–E3), sucrose (F1–F3), maltose (G1–G3) and sugar mixture (H1–H3). Amplifications: A1–H1, ×5000; A2–H2, ×50 000. The diameters of fibrils in bundles were calculated separately.

The scanning electron microscopy (SEM) micrographs and the fibril diameters of the BNC are shown in Fig. 3. All BNC samples exhibited a three‐dimensional reticulated structure consisting of ultrafine cellulose fibrils. The fibril diameter of BNC from media based on glucose, maltose and the sugar mixture (19–22 nm) was significantly (*P* < 0.05) lower than that of BNC from media based on the other carbon sources (27–31 nm). As the use of media with glucose, maltose or the sugar mixture had higher volumetric BNC productivity (Fig. 3), it is a possibility that high volumetric productivity and low fibril diameter are related. The average fibril diameter of 19–31 nm of BNC in the current work (Fig. 3A3–H3) is comparable to that of 30–40 nm from strain ATCC 23770 as reported previously (Cavka *et al*., 2013).

### FTIR spectra of BNC

**Figure 4:**
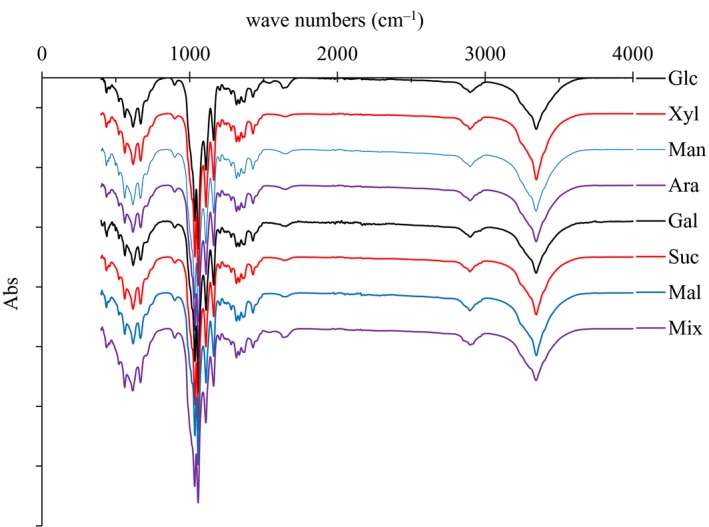
Fourier‐transform infrared spectra of bacterial nanocellulose produced from eight carbon sources.

Fig. 4 displays the FTIR spectra of BNC produced in culture media based on the eight carbon sources. The peaks at around 1158 cm^−1^ are characteristic for the glycosidic links of cellulose (Dokken *et al*., 2005). The peaks at 895 and 1427 cm^−1^ can be assigned to deformation of anomeric CH and CH_2_ bending in cellulose respectively (Kataoka and Kondo, 1999). The peaks at 710 and 750 cm^−1^ are characteristic for cellulose I_*β*_ and cellulose I_*α*_, respectively (Kataoka and Kondo, 1999). The peaks at around 3350 cm^−1^ can be assigned to the vibration of OH in cellulose (Lu and Jiang, 2014). The peak at 2900 cm^−1^ can be assigned to the C‐H stretching vibration in cellulose (Lu and Jiang, 2014). These results are consistent with the characterization of the specimens as cellulose, and, as judged by FTIR spectroscopy, there was no significant difference among the BNC preparations from the culture media with the eight carbon sources.

### Viscometric degree of polymerization of BNC

**Figure 5:**
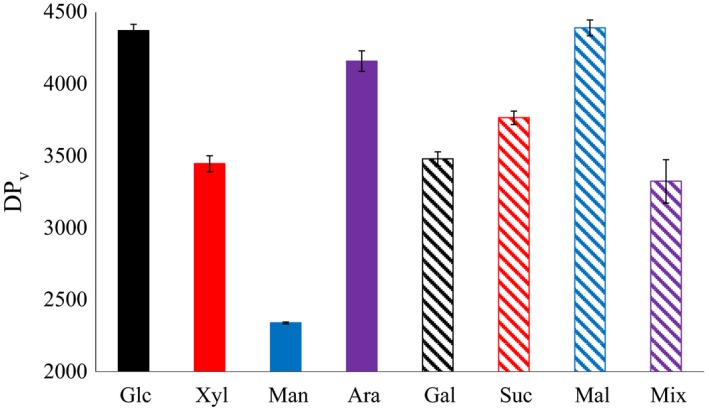
Average viscometric degree of polymerization of bacterial nanocellulose produced from eight carbon sources.

The average viscometric degree of polymerization (DP_v_) of the BNC produced from the eight carbon sources is shown in Fig. 5. The DP_v_ values of BNC from cultures with glucose and maltose were similar (4350–4400) and significantly (*P* < 0.05) higher than the DP_v_ values for BNC from the other cultures. The DP_v_ of BNC from cultures with mannose (2340) was far lower than the DP_v_ of BNC from cultures with the other carbon sources (significant difference with *P* < 0.05). The high DP_v_ of BNC from cultures with glucose and maltose and the low DP_v_ of BNC from cultures with mannose coincided with differences in BNC productivity and BNC yield for cultures based on these sugars. The DP_v_ of BNC from cultures with the sugar mixture was on an intermediate level (Fig. 5).

Compared with other studies, the DP_v_ of the BNC in the current investigation was relatively high, except for the BNC from cultures with mannose‐based medium (Fig. 5). Using *K. xylinus* ATCC 23769 and measuring DP with a similar method as in the current investigation, Shibazaki *et al*. (1997) reported a DP_v_ of 2000 for BNC and 2280 for cotton linter.

The difference in DP_v_ of BNC from cultures with media based on different sugars is consistent with previous reports. Shi *et al*. (2013) found that the DP of BNC from cultures with xylose‐based medium was much lower than that of BNC from cultures with glucose‐based medium. However, the current investigation is the first that shows the effects of as many as eight carbon sources on the DP_v_ of BNC, and also the first that shows that BNC from cultures with medium based on a sugar mixture can have lower DP_v_ than BNC from cultures with medium based on individual sugars.

### X‐ray diffraction spectra of BNC

**Figure 6:**
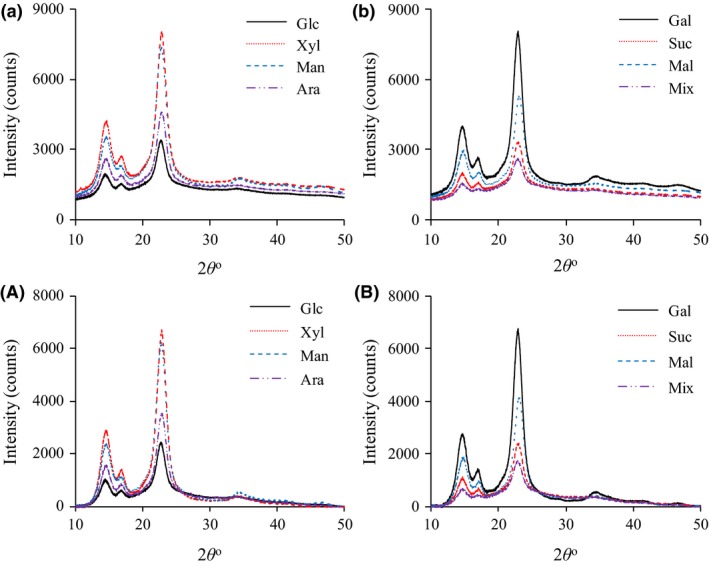
X‐ray diffraction spectra of bacterial nanocellulose produced from eight carbon sources (a and b: spectra before baseline subtraction; A and B: spectra after baseline subtraction).

Fig. 6 shows XRD (X‐ray diffraction) spectra of BNC produced from cultures with media based on the eight carbon sources. Data on the crystal structure are shown in Table 1. The crystallinity indices of BNC from cultures with galactose, xylose and mannose (76–78%, shown in bold) were the highest among the BNC preparations, whereas the crystallinity index of BNC from cultures with the sugar mixture (54%) was the lowest. The higher crystallinity indices of BNC from galactose, xylose and mannose coincide with low productivity, low yield and low DP_v_. Regarding the difference in Bragg angle between the peaks of plane (101) and plane (10i), the BNC from cultures with medium based on the sugar mixture had the highest value, while the BNC from cultures with mannose‐based medium had the lowest value. This suggests that the BNC from cultures with the sugar mixture had the highest content of cellulose I_*α*_ and that the BNC from cultures with mannose‐based medium had the lowest content of cellulose I_*α*_ (Watanabe *et al*., 1998). The crystallite size of the different crystallite planes of BNC from cultures with different carbon sources varied (Table 1). BNC from cultures with sucrose‐based medium had the largest crystallite size in all planes (Table 1, in bold).

**Table 1:** Crystal properties of BNC produced from cultures with eight different carbon sources

	Crystallinity index (%)	Difference in Bragg angle (degrees)^a^	Crystallite size (nm)			
			*L* (101)	*L* (10i)	*L* (002)	*L* (040)
Glucose	61	2.34	4.27	4.32	4.49	4.99
Xylose	**77**	2.29	3.95	4.17	4.53	4.84
Mannose	**76**	**2.17**	4.24	4.28	4.45	4.98
Arabinose	67	2.28	5.07	5.13	5.32	5.96
Galactose	**78**	2.32	4.73	3.40	5.58	2.39
Sucrose	61	2.39	**5.17**	**5.24**	**5.43**	**6.04**
Maltose	71	2.33	4.14	4.37	4.76	5.08
Sugar mix	54	**2.43**	4.21	4.26	4.42	4.91

**a**. Difference in Bragg angle between peaks of plane (101) and plane (10i) in Fig. 6A and B.

Compared with other studies (Watanabe *et al*., 1998; Lu and Jiang, 2014), the BNC in the current study exhibited similar crystallinity index but smaller crystallite size. For example, Watanabe *et al*. (1998) found that the crystallinity index of BNC produced from static cultures with fructose‐based medium was 71% and that the crystallite size of plane (101) was 7.4 nm. Krystynowicz *et al*. (2002) found that the crystallinity index of BNC produced from static cultivation of *K. xylinus* E25 in glucose‐based medium was 50%.

There are few comparisons of the crystallinity of BNC produced in media based on different sugars. Molina‐Ramírez *et al*. (2017) found that the crystallinity index of BNC from cultures with sucrose‐based medium was slightly higher than that of BNC from cultures with glucose‐based medium. The current study, which covers BNC production from eight carbon sources and determination of both crystallinity and crystallite size, suggests that sugars that give low BNC productivity and low BNC yield could give high BNC crystallinity. Furthermore, the results suggest that a sugar mixture, as in a lignocellulosic hydrolysate, would give low BNC crystallinity but high BNC productivity and yield.

### Thermogravimetric analysis of BNC

**Figure 7:**
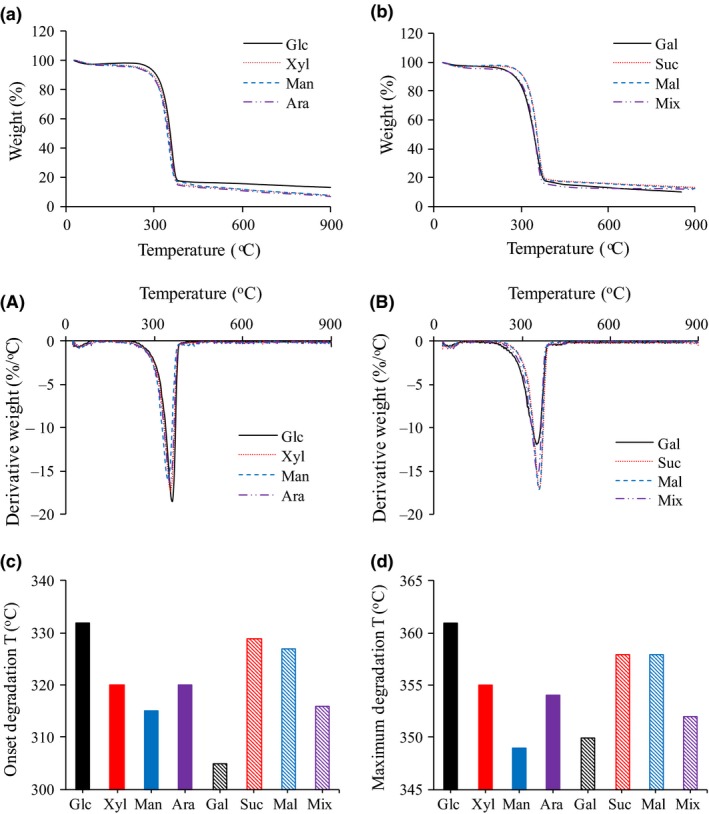
The figure shows results from thermogravimetric analysis (**a** and **b**), differential thermogravimetry (**A** and **B**), the corresponding onset degradation temperature (**c**) and the temperature at the maximum degradation rate (**d**) for bacterial nanocellulose produced from eight carbon sources.

**Figure 8:**
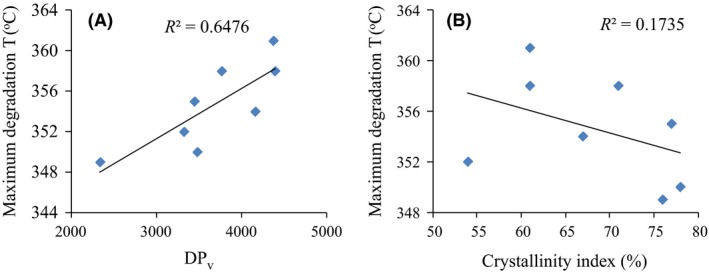
Correlation between degree of polymerization_v_ (A) and crystallinity index (B) of bacterial nanocellulose (BNC) and the temperature at the maximum degradation rate of BNC.

The results of thermogravimetric analysis (TGA) are shown in Fig. 7a and b, whereas results from analysis with differential thermogravimetry (DTG) are shown in Fig. 7A and B. The corresponding onset degradation temperature and temperature at maximum degradation rate for the BNC samples are shown in Fig. 7c and d. The BNC from cultures with glucose‐based medium had the highest onset degradation temperature, while the BNC from cultures with galactose‐based medium had the lowest. Regarding the temperature at the maximum degradation rate, the BNC from cultures with glucose‐based medium still had the highest value (361°C), whereas BNC from cultures with mannose‐based medium had the lowest value (349°C). Plots of the DP_v_ and the crystallinity index of the BNC against the temperature at the maximum degradation rate (Fig. 8A and B) indicated that the temperature at the maximum degradation rate of the BNC exhibited a weak positive correlation with the DP_v_ (*R* ^2^ = 0.65), whereas there was no correlation for the crystallinity index (*R* ^2^ = 0.17). However, it should be noticed that the correlation between the temperature at the maximum degradation rate and the DP_v_ could be more complicated than a simple linear relationship.

The thermal stability of BNC has been analysed previously. Generally, the results of previous studies have been similar to those of the current investigation, except that the thermal stability of BNC from cultures with a sugar mixture has not been thoroughly investigated before. Chandrasekaran *et al*. (2017) found that BNC from *K. xylinus* ATCC 11142 had a maximum degradation rate at around 300°C. The thermal stability of BNC produced by *K. xylinus* PTCC 1734 using media with mannitol or sucrose was analysed, and it was reported that the decomposition of the BNC samples resulted in a significant weight loss (70–80%) at around 360–390°C (Mohammadkazemi *et al*., 2015). The difference in thermal stability of the BNC samples in the current study could be attributed to structural differences in BNC produced in cultures with different sugars.

In conclusion, this investigation elucidated the significant impact of the carbon source in the culture medium on the productivity, structure and properties of BNC. Whereas some carbon sources gave high BNC productivity, yield and DP_v_, others gave high BNC fibril diameter and high crystallinity. The sugar mixture resembling a lignocellulosic hydrolysate gave relatively high BNC productivity and BNC yield on consumed sugar, but relatively low DP_v_, crystallinity and thermal stability. Further investigations are needed to clarify the metabolic pathways of the different sugars and how they contribute to different BNC productivity and quality. The large difference in consumption rate between the two industrially important disaccharides sucrose and maltose needs to be better understood. The current study suggests that the yield and DP of BNC produced from cultures with mannose need to be improved, if BNC is to be produced using feedstocks with a high content of mannan, such as softwood. Furthermore, the low BNC yield from culture medium based on xylose suggests that research on xylose utilization by *K. xylinus* warrants further attention, especially as most lignocellulosic feedstocks contain considerable amounts of xylan.

## Experimental procedures

### Microorganism and chemicals

*Komagataeibacter xylinus* (formerly *Gluconacetobacter xylinus*) ATCC 23770 was obtained from the American Type Culture Collection (Manassas, VA, USA) and was maintained as glycerol stocks at −80°C. *K. xylinus* ATCC 23770 was used as it is a widely studied BNC‐producing bacterial strain (Thompson and Hamilton, 2001; Hong and Qiu, 2008; Cavka *et al*., 2013). The sugars used for preparing media, i.e. glucose (glc), xylose (xyl), mannose (man), arabinose (ara), galactose (gal), sucrose (suc) and maltose (mal), were of spectral grade. The peptone was from Merck KGaA (Darmstadt, Germany). The yeast extract was from VWR Chemicals (Radnor, PA, USA). Deionized water was used in all the experiments.

### Culture media

The seed culture medium used for preparing inocula contained 25 g l^−1^ glucose, 5 g l^−1^ peptone and 3 g l^−1^ yeast extract. The initial pH was adjusted to 5.0 by using an 8 M aqueous solution of NaOH.

In the comparison of the eight carbon sources, the culture medium contained 40 g l^−1^ sugar, 10 g l^−1^ peptone and 6 g l^−1^ yeast extract. Glucose, xylose, mannose, arabinose and galactose were included as they are common in lignocellulosic hydrolysates. Sucrose is found in sugar industry by‐products, such as molasses and bagasse. Maltose is obtained from hydrolysis of starch. The 40 g l^−1^ carbon source in the sugar mixture consisted of 24 g l^−1^ glucose, 9 g l^−1^ xylose, 4 g l^−1^ mannose, 2 g l^−1^ arabinose and 1 g l^−1^ galactose. This mixture was designed to resemble a hydrolysate of lignocellulose, which is an abundant source of carbohydrate in nature (Humbird *et al*., 2011; Guo *et al*., 2013; Domanski *et al*., 2016; Normark *et al*., 2016). The final pH was adjusted to 5.0 using the 8 M aqueous solution of NaOH. The volume of medium in each 250 ml flask was 100 ml.

### BNC production using different carbon sources

Seed cultures were prepared, and inoculation was conducted as previously reported (Chen *et al*., 2018a,b). Static cultivations of *K. xylinus* in 250 ml flasks were performed at 30°C. Samples consisting of 1 ml culture broth were taken every 4 days for determination of pH and residual sugar. After 11 days, the BNC was harvested by centrifugation at 12 000 *g*. Triplicate cultivations were performed, and mean values are reported. The metabolic mechanisms behind the differences in BNC production were not within the scope of the current study. The current study was designed to show the differences with regard to the quantity and quality of the BNC among culture media based on different sugars.

Due to current limitations in analytical techniques, the biomass evolution was not followed during the cultivations. In static cultivation, *K. xylinus* cells are mostly embedded in the BNC membrane. Currently, there is no convenient and reliable method to count cells during static cultivation (Zou *et al*., 2017). More work is needed in the future to develop analytical techniques for measuring biomass evolution in static cultures.

The harvested crude BNC was washed five times at 80°C, each time for four h, using a 0.1 M aqueous solution of NaOH. After that, it was washed five times with deionized water at 80°C, each time for four h. The washing with the NaOH solution was performed a larger number of times and for longer period of time than in most other studies, in which washing was done only once and for no more than one h (Watanabe *et al*., 1998; Bae *et al*., 2004). Extensive washing would give a purer product, but might negatively affect the yield. The purified BNC was then freeze‐dried and weighed to calculate BNC productivity and yield. After washing and weighing, the quality of freeze‐dried BNC was analysed.

### Measurement of sugar

Analysis of the sugars was performed by using high‐performance liquid chromatography (HPLC). A Bio‐Rad Aminex HPX‐87P Column (7.8 × 300 mm) was used in an Agilent 1260 Infinity series system (Agilent, Santa Clara, CA, USA) equipped with a 1260 series diode array and multiple wavelength detector (DAD). Elution was performed with isocratic flow of high‐quality nano‐pure water. The flow rate was 0.6 ml min^−1^, and the column temperature was set to 80°C. Agilent software was used for data analysis. The sugar consumption ratio was calculated as follows:

$$\begin{array}{c} \text{Sugar consumption ratio}=[1-\\ (\text{final sugar concentration/initial sugar concentration})]. \end{array}$$

### Characterization of BNC from eight carbon sources

Analyses of BNC using SEM, FTIR spectroscopy and DP_v_ were conducted according to methods described previously (Chen *et al*., 2017, 2018a,b). XRD analysis was performed with an AXS d8 Advance X‐ray diffractometer (Bruker, Germany) using Cu Kα‐radiation (40 kV, 40 mA) with a line‐focus tube and with a two‐dimensional detector. The scan range was 2*θ* 10–50° with a step size of 0.005°. Calculations of the crystallinity index (expressing the relative degree of crystallinity) without any baseline subtraction of the XRD spectra were based on the empirical method reported by Segal *et al*. (1959). The apparent crystallite sizes (*L*) of the crystallographic planes of (101), (10i), (002) and (040) were calculated from the corresponding Bragg angle in baseline‐subtracted spectra as previously reported (Chen *et al*., 2018a,b).

Thermogravimetric analysis was carried out by using a TG‐209‐F1 Libra gravimetric analyser (Netzsch, Selb, Germany). BNC samples (3–5 mg) were weighed and loaded into an alumina pan, and were heated from 25 to 900°C with a heating rate of 10 K min^−1^ under a flow of nitrogen gas (250 ml min^−1^).

## Conflict of interest

None declared.
